# Recent Advances and Future Challenges in Modified Mycotoxin Analysis: Why HRMS Has Become a Key Instrument in Food Contaminant Research

**DOI:** 10.3390/toxins8120361

**Published:** 2016-12-02

**Authors:** Laura Righetti, Giuseppe Paglia, Gianni Galaverna, Chiara Dall’Asta

**Affiliations:** 1 Department of Food Science, University of Parma, Parco Area delle Scienze 95/A, Parma 43124, Italy; laura.righetti1@studenti.unipr.it (L.R.); gianni.galaverna@unipr.it (G.G.); 2 Center of Biomedicine, European Academy of Bolzano/Bozen, Via Galvani 31, Bolzano 39100, Italy; beppepaglia@gmail.com

**Keywords:** modified mycotoxins, high resolution mass spectrometry, ion mobility spectrometry

## Abstract

Mycotoxins are secondary metabolites produced by pathogenic fungi in crops worldwide. These compounds can undergo modification in plants, leading to the formation of a large number of possible modified forms, whose toxicological relevance and occurrence in food and feed is still largely unexplored. The analysis of modified mycotoxins by liquid chromatography–mass spectrometry remains a challenge because of their chemical diversity, the large number of isomeric forms, and the lack of analytical standards. Here, the potential benefits of high-resolution and ion mobility mass spectrometry as a tool for separation and structure confirmation of modified mycotoxins have been investigated/reviewed.

## 1. Introduction

The presence of food and feed contaminants, in particular secondary fungal metabolites, has become of increasing concern for consumers and producers. Indeed, more than 400 mycotoxins with widely different chemical structures have been identified so far, and their number is expected to increase further due to climate changes [1]. Extreme weather conditions are increasingly affecting the mycotoxin map in Europe and also world-wide, leading to an unpredictability of the range of mycotoxins occurring in food crops. In addition, it is already well known [2] that plants and other living organisms (i.e., fungi, bacteria, mammals) can alter the chemical structure of mycotoxins as part of their defense against xenobiotics, and thus contribute to further increase the wide spectrum of possible occurring contaminants. Mycotoxins, indeed, may undergo [2,3] phase-I, and phase II metabolism, involving in the former, chemical reactions such as oxidation, reduction and hydrolysis, and in the latter conjugation with amino acids, glucoses, sulfate groups and glutathione. All these modifications significantly change the chemical structure of the parent compounds.

According to the recent EFSA opinion [3], which aimed to harmonize the terminology across the scientific community, all these metabolites are referred to as “modified mycotoxins” [4], being structurally altered forms of the parent mycotoxins. The definition also covers the metabolites originating after thermal/ process degradation. Modified mycotoxins may co-occur as contaminants in addition to parent compounds in food and feed; so far modified forms for trichothecenes, zearalenone (ZEN), fumonisins, *Alternaria* toxins and ochratoxin A (OTA) have been identified. However, up to now their occurrence in naturally infected cereals has been exclusively confirmed for deoxynivalenol (DON), ZEN and fumonisins [5,6,7]. In fact, the lack of analytical standards and reference materials, has substantially complicated their identification, partially restraining research progress in the field as well.

The metabolic conjugation with polar molecules is commonly considered as an inactivation reaction, because the aglycone usually loses its biological activity. However, the possible hydrolysis of modified mycotoxins back to their toxic parents during mammalian digestion raises toxicological concerns [8] and thus the requirement for their detection. Modified mycotoxins were originally considered “masked” [9] since they may elude conventional analysis because of impaired extraction efficiency caused by increased polarity when a less polar solvent is used for the extraction of non-modified mycotoxins. Moreover, the changed physicochemical properties of their molecules lead to modified chromatographic behavior and due to the lack of analytical standards they are currently not routinely screened.

All of these effects may lead to a potential underestimation of the total mycotoxin content of the sample. Therefore, monitoring the presence of these potentially hazardous metabolites remains one of the main tasks for ensuring food safety and human/animal health.

In this frame, liquid chromatography coupled with mass spectrometry has represented the golden standard for at least a decade. This review aims, therefore, at pointing out the possible advantage of innovative MS techniques in mycotoxin analysis, and to highlight the improvements still needed to meet the future challenges in the field.

## 2. From Targeted LC-MS/MS Determination to Untargeted HR-MS Analysis

In the field of residues and contaminants, analytical methods used for surveillance purposes must ensure optimal sensitivity and accuracy. For this reason, chromatographic analysis coupled to fluorescence or UV detection was the reference techniques for many decades. Over the last two decades, the LC-MS/MS platform became the method of choice, in particular for its ability to allow the development of multi-residue and multi-class methods [10,11,12,13,14]. First attempts were aimed at quantifying a single mycotoxin, but later on the research moved to the simultaneous determination of multiple mycotoxins, leading to the development of the so-called multi-toxin methods for quantitative as well as screening purposes [13,14,15].

The current “golden standard” in routine food safety control is represented by unit resolution tandem mass spectrometric analyzers such as triple quadrupole (QqQ) [16,17], mainly because this technique ensures analytical parameters that easily meet quality criteria required by law [18,19,20]. Multiple reaction monitoring (MRM) has been traditionally selected for mycotoxin analysis, monitoring in parallel quantitative and qualitative ion transitions, providing both sensitivity and selectivity. Achieved limits of quantification (LOQs) and detection (LODs) usually match regulatory requirements for the official control method for contaminants. The first low-resolution multi-toxins method was developed in 2006 [13] for the quantitative determination of 39 parent and modified mycotoxins. Among the modified mycotoxins, the methods included 3Ac-DON, 15Ac-DON, DON3Glc, ZEN14Glc, ZEN14Sulf, and hydrolysed FB1. More recently [21] a method for the simultaneous quantification of both parent and modified *Alternaria* mycotoxins in cereals based food, including alternariol (AOH), AOH3Sulf, AOH3Glc, alternariol-methyl ether (AME), AME3Sulf, and AME3Glc was developed. *Alternaria* modified mycotoxins were in-house synthesized, since they are not commercially available yet. In addition, the applicability of the developed methods has been demonstrated by analysis of a variety of naturally contaminated cereals and real-life samples purchased on the market [7,13,21,22,23].

Despite having become a well-established technique, the QqQ method set-up is time-consuming when aimed at determining a large number of substances. Likewise, this technique presents limitation on the number of compounds that can be analyzed in one run. In addition, only targeted analytes can be detected, making necessary the use of an analytical standard, which is a critical issue in the modified mycotoxins field.

Thus, with the introduction of benchtop high resolution mass spectrometers (HRMS), such as Time-of-Flight (ToF) and Orbitrap, full-scan techniques started to be investigated as a complementary approach for the triple-quadrupole-based methods on the basis of increased resolution power and detectability. In addition, the use of LC coupled to HRMS offers some advantages over QqQ, since the acquisition of high resolution full scanned data permits the combination of target analysis with screening of non-target compounds, novel compound identification, and retrospective data analysis.

The increasing popularity of HRMS is mainly due to the advantages of using the full-scan acquisition mode [10,24,25] with high sensitivity, combined with high resolving power (up to 100,000 FWHM) and accurate mass measurement (<5 ppm). Moving from low resolution MS to high resolution should improve in principle specificity, although this is difficult to be transferred to a superior performance of the target analysis when moving from LC-MS/MS to HRMS methods, since ion suppression/enhancement phenomena due to the matrix may occur in both approaches. A possible approach to cope with the matrix effect is represented by stable isotope dilution assay (SIDA) [26]. Multi-mycotoxins methods applying this technique have been developed [27,28], also thanks to in-house synthesis of labeled isotopologue mycotoxin standards, including isotopologues of modified forms (i.e., DON3Glc, 3Ac-DON, 15Ac-DON) [29]. Authenticity and method performance have been demonstrated by analyzing naturally contaminated samples, such as malt, beer and maize [30]. Despite ion suppression phenomena, the enhanced selectivity and sensitivity provided by HRMS allow the development of methods that cover a wide range of compounds with different physicochemical properties, as demonstrated by Dzuman and coworkers [31] who developed an LC-HRMS method for the detection of 323 pesticides, 55 mycotoxins, and 11 plant toxins.

The major advantage of using HRMS over MS/MS techniques is actually due to the possibility to perform retrospective data analysis [32], thus enabling the possibility to reconsider analytical results for stored data. The measurement of accurate MS and MS/MS spectra (resolution <5 ppm) allows the determination of compounds without previous compound-specific tuning, to carry out retrospective analysis of data, and to perform structural elucidation of unknown or suspected compounds. This is particularly worth noting when modified mycotoxins are considered, especially if combined toxicological effects are in the pipeline.

## 3. Use of HRMS Methods for Targeted Quantification of Natural Toxins

From a quantitative point of view, as main advantage, a full-scan technique allows the extraction from the HRMS full scan data of a theoretically unlimited number of analytes without any compromise regarding the resulting detectability. Generic tuning setting can be used, without the need for optimizing parameters for each analyte.

Although the use of HRMS in food is very recent, over the last few years there was a significant increase in the number of studies reporting LC-HRMS-based approaches for targeted quantitative analysis of residues and contaminants in complex food matrices [10,24,33,34], most of them using a multi-contaminant approach [11,12,31,32].

Among the classes of food contaminants, the possible use of HRMS-based methods for natural toxins was successfully applied to the analysis of a large variety of samples from the market, as recently reviewed by Senyuva et al. [35]. In general, the collection of exact *m*/*z* values, HRMS/MS spectra and retention time allow the build up of (myco)toxins spectral libraries potentially sharable between Q-Orbitrap instruments. As an example, Ates et al. [36] created a database containing empirical formulae, polarity, fragment ions (up to five), and retention times for 670 plant and fungal metabolites. The library was validated by correct identification of known mycotoxins in proficiency test materials, and then applied to the screening of emerging mycotoxins in cereal samples from the market.

While the use of HRMS for the quantification of one or few analytes does not pose any significant advantage compared to MRM-based methods, multi-contaminant methods seem to be the most promising approach for food and feed surveillance in the coming years. It must be underlined, however, that the validation steps required for HRMS-based methods does not differ from those applied for QqQ methods. Moreover, sample preparation (i.e., extraction, enrichment, clean up, chromatographic separation) still remains a crucial step to reduce ion suppression phenomena, and ensure the required specificity and detectability.

Concerning mycotoxins, the possible set up of HRMS multi-toxin methods has been increasingly exploited in recent years, especially for monitoring the co-occurrence of regulated and emerging mycotoxins. In this frame, the potential capability of HRMS to return a full picture of the pool of modified mycotoxins in a selected food may represent the basis for future studies of combined toxicity.

However, it should be mentioned that only five modified mycotoxins have been included so far in multi-toxin HRMS quantitative methods, since analytical standards are available on the market only for DON3Glc, 3Ac-DON, 15Ac-DON, as well as α and β zearalenol (ZEL) [31]. Other modified forms have been included in semi-quantitative or screening methods, based on in-house prepared reference compounds, usually obtained by chemical/enzymatic synthesis or isolation from natural sources. In other studies, conjugates are semi-quantified on the base of the parent compounds. Although inaccurate, in case of novel compounds, when neither commercial calibrants nor in-house synthesized standards are available, this assumption allows for a rough semi-quantitation of the novel conjugate compared to the parent form. For instance, zearalenone biotransformation to zearalenone malonyl-glucoside in wheat was estimated by assuming that both the parent and the modified form had the same response during MS ionization [37]. As an alternative, the formation of DON-oligoglucosides during malting and brewing processes was expressed as their molar ratio to DON [38].

In consideration of the lack of commercial standards, HRMS actually provides more qualitative than quantitative benefits to modified mycotoxin analysis.

## 4. Use of HRMS Non-Targeted Screening Methods for Natural Toxins

In consideration of the possible collection of full-scan spectra, a theoretically unlimited number of compounds from different classes may be detected simultaneously by HRMS at low concentration level. Therefore, HRMS is often used for non-targeted screening of unknowns, since compound to be monitored should not be established in advance. As a general remark, when natural toxins are considered, it must be noted that unknown compounds should be better defined as “expected unknowns” and “unexpected unknowns”, the former being modified forms of natural compounds that can be anticipated on the basis of biological pathways, and the latter novel compounds never described before. In addition, known compounds may occur in unexpected biological matrices, thus representing an unexpected known analyte. Examples of this definition are collected in Table 1.

In a more general meaning, a “non-targeted” analysis could be described as a screening against a large database of compounds, or a retrospective analysis of a dataset for compounds not specifically anticipated. This approach usually leads to a list of potential contaminants occurring in a sample that should be further confirmed by targeted analysis. The applicability of HRMS as a non-targeted approach indeed is based on the screening of an accurate mass of both precursor and fragments ions in one single run, by using data-independent analysis (DIA), without monitoring any preselected parent ions and based on general settings. This permits retrospective data analysis from the recorded HR-full-scan spectra; consequently, the presence of ‘newly discovered’ mycotoxins can be investigated with the data of prior-analyzed samples without the need for analytical standards.

**Figure 1:**
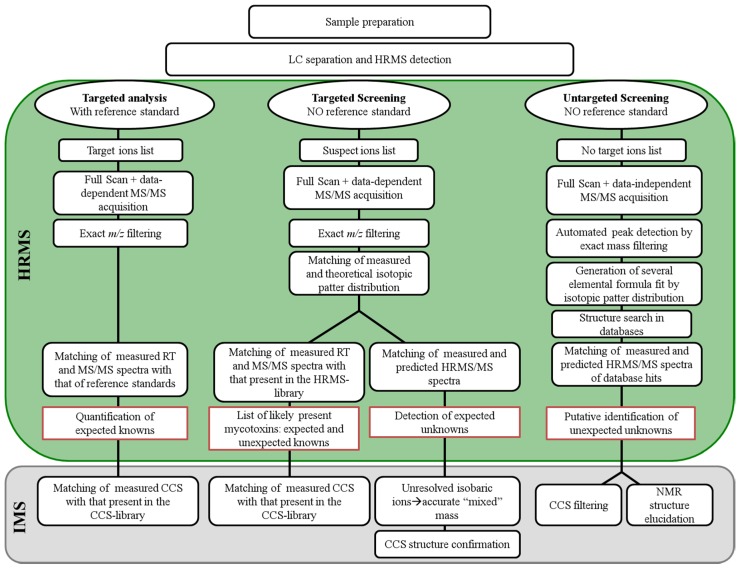
Workflow for targeted analysis with mycotoxin reference standards, targeted screening without analytical standards, and untargeted screening for unexpected unknowns (adapted from Krauss et al. [25]). CCS = Collision Cross Section.

As can be seen below (Figure 1), the untargeted workflow usually moves from seeking the exact masses of a list of compounds in the full scan spectra, to generating molecular formula, and comparing theoretical and experimental isotopic patterns and fragment spectra with those collected in the reference library, or available in on-line databases. Therefore, the full process can be described as the search of a limited number of compounds (those reported in the library) in an unlimited dataset (the stored data).

Screening methods are usually based on a generic sample preparation (i.e., QuEChERS extraction procedure) [15,31], and thus data collected allow in principle the retrospective review of any potential compounds of interest. Nonetheless, the final detection and quantification of targeted compounds may be negatively affected by interferences from the matrix. In some cases, co-elution may impact on the mass accuracy by causing ion suppression. This may lead to the missing of suppressed compounds during the automated filtering process. Therefore, the improvement of sample preparation as well as the fit-for-purpose adjustment of software parameters are of great relevance for the analysis.

A comprehensive description of the systematic workflow for quantitative target analysis, targeted screening of listed compounds, and untargeted screening of unknowns was reported by Krauss et al. [25].

Considering the large number of data generated by using HRMS, reliable bioinformatics tools for processing untargeted data are needed, as well as software packages for an automated compound detection. Therefore the development of a reliable bioinformatics workflow, providing a software solution from features extraction to unknown identification data is fundamental. The selection of relevant compounds from a large data set still represents the bottleneck [51]. Recently developed approaches show that statistical analyses, in combination with untargeted screening, are useful methods to preselect relevant compounds [52]. In addition, there are different software tools that allow peak deconvolution and the removal of background noise [25] by comparing different chromatograms. As an example, the use of a blank matrix as a control sample can substantially reduce the number of compounds to be screened by the software algorithm.

At this point, several elemental composition formula may be predicted from an accurate mass measurement using MS trademark packages. Subsequently, molecular formula candidates and MS/MS pathway may be jointly investigated by structure elucidation software. Nowadays, HRMS qualitative software tools are linked with on-line databases (i.e., Pubchem, Metlin) providing a comparison of accurate mass, isotopic pattern, and MS/MS fragment ions. In the end, the users may find a percentage of matches, which are interconnected with the unknown compound. However, it should be noted that the search for unknowns in on-line databases is limited to the recorded spectra of available reference standards. In this frame, a big step forward has been achieved with computer-based tools based on in silico strategies [51,52], since the unknown chemical structures may be putatively confirmed by matching measured and computational predicted MS/MS fragmentation.

## 5. Use of HRMS Methods for Structural Identification of Unknowns

**Figure 2:**
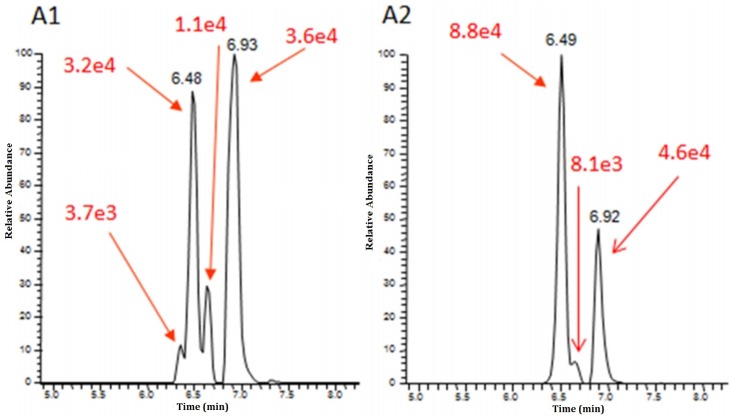
Extracted ion chromatogram for DON di glucosides determined in malt (**A1**) and beer (**A2**) by using HILIC phase chromatography coupled to HRMS (Orbitrap). (Reproduced with permission from [29], copyright (2016) American Chemical Society).

Another relevant application of HRMS is the structural identification of unknown compounds, i.e., novel compounds identified for the first time in the considered matrix. Although the unequivocal structural elucidation of compounds still requires ^1^H- or ^13^C NMR spectroscopy, in most cases there is already sufficient information to tentatively annotate and identify the unknowns. This is the case, for instance, of expected unknowns, modified mycotoxins where the possible modification pattern carried out by plants or microbes is well-established. As an example, the identification of new trichothecene conjugates such as FUSX-glucoside and NIV-glucoside [45], or acetyl-T2 [48,53] was an extrapolation of existing knowledge. The tentative identification of mycotoxin gluco-conjugates based on accurate mass, isotopic pattern distribution, and MS/MS fragmentation is indeed feasible. Accurate mass can be theoretically calculated and MS/MS fragments may be quite easily predicted since they generally lead to the loss of the glucosidic unit [M–H–glucose]^-^ and thus the detection of the aglyconic form. More challenging is the elucidation of the binding position of the sugar unit. As suggested by Dall’Asta et al. [54] the binding position in the DON molecule distinctly influences the stability of the molecular ion, thus leading to different fragments. In particular, the ion corresponding to [M–H–CH_2_O]^−^ is reported to be characteristic of all 3-substituted trichothecenes; however that is not true for the other classes of mycotoxins. By following this approach, also DON-oligoglycosides [38] were identified in malt and beer samples. Therefore, the molecular formula and exact masses of DON-di, tri, and tetra glucosides can be easily calculated by adding glucose units corresponding to 162.0523 Da, as summarized in Table 2, and thus many putative structures can be predicted. As a result, nine possible molecular structures were hypothesized for DON-di-Glc, corresponding both to di-glucoside conjugates, with one molecule of glucose conjugated to each of the hydroxyl groups of DON or oligosaccharides. Although chromatographic separation was optimized changing from a reverse phase to an HILIC column, isobar separation was not achieved. In addition, also considering the peaks that are not baseline-resolved, no more than four out of nine peaks were detected both in malt and beer samples, as is depicted in Figure 2.

**Figure 3:**
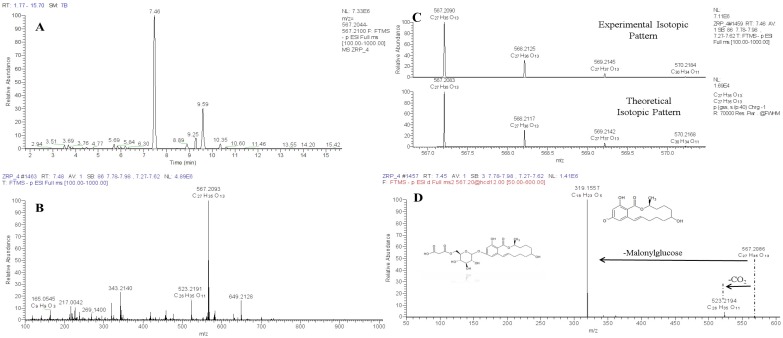
ZEL-MalGlc putative identification steps: UHPLC-Q-Exactive (**A**) full scan extracted ion chromatogram (resolving power 70,000 FWHM, extraction window 5 ppm); (**B**) molecular formula assignment of parent ion; theoretical and experimental isotopic pattern comparison (**C**,**D**) high resolution fragmentation pathways obtained by using DDA acquisition.

As well as gluco-conjugates, also mycotoxins conjugation with malonyl-glucoside frequently occurs. So far, occurrence of T2, HT2, ZEN, α and β ZEL, and DON malonyl-glucosyl (MalGlc) derivatives have been reported in artificially infected samples [44,47,48,53]. The extracted ion chromatogram (EIC) of tentative HT2-malonyl-glucoside showed two peaks that may result from the conjugation of malonic acid to different hydroxyl groups of glucose (four possible positions) or from conjugation of malonyl-glucoside to the two different position of the HT2 hydroxyl groups [39]. As an example, the ZEL-MalGlc HRMS putative identification workflow is depicted in Figure 3.

Thus, also in these examples, it was not possible to achieve a high degree of certainty for the identification of expected unknowns and thus the authors concluded that NMR analysis was required. In addition, putative co-eluting isomers having the same *m*/*z* value can be present in the sample, considering the high number of theoretical structure compared to the detected peaks.

As far as modified mycotoxins, the metabolic modification occurring in plants usually follows phase I and phase II patterns. Therefore, the possible modification can be theoretically anticipated, and a list of expected unknowns can be used for peak annotation. As an example, a list of possible phase I and phase II modifications is reported in Table 2.

Several expected unknowns of mycotoxins have been tentative identified so far thanks to the use of HRMS [55]. When only one isomer configuration is possible, the recorded HRMS/MS spectra allow a satisfactory compound structure elucidation. However it should be stated that many mycotoxins are characterized by more than one hydroxyl group, that can be further increased by phase I metabolism. As a result, conjugation reactions may take place on several substituent groups and thus separation and characterization of the different isomers still remain challenging.

However, although it is frequently based on the search of expected unknowns, structural elucidation in complex samples is limited by the non-specific nature of the electrospray ionization process. It has been stated, indeed, that full scan spectra may contain up to 90% of noise compared to low concentration metabolites [56]. In such cases, the use of stable isotope labeling (SIL) may successfully assist metabolic profiling [57]. The software-driven correction of isotope pattern abundance errors resulted in better identification rates of the molecular formulas. In particular, artifacts generated by solvents, matrix background or noise, can be filtered out, enabling better detection and annotation of novel and unexpected compounds.

Recently, the in vivo SIL approach was exploited for the identification of *Fusarium* toxin metabolites in grains, based on the comparison of natural and ^13^C-labelled patterns of metabolites showing identical chromatographic behavior but different (shifted) MS spectra [48,58]. The spectra comparison and metabolite identification was supported by the use of a dedicated software [59]. This powerful approach may simplify the identification of novel unexpected unknown compounds (i.e., feruloyl-T2 [48]) and successfully support the metabolic fingerprint.

## 6. Advantages and Challenges of HRMS in Modified Mycotoxin Analysis

When natural toxins are considered, the advantages derived from a qualitative use of HRMS overcome those obtained from a quantitative analysis. Retrospective data analysis, expected unknown screening, and novel compound identification are actually features that may strongly improve research in this field in the very near future. This is especially interesting in the field of emerging and modified mycotoxins, when analytical standards, calibrants, and reference materials are not commercially available. In addition, the urgent trend to base risk assessment on the combined toxicity derived from a pool of contaminants instead of a single compound, makes necessary the collection of (co)occurrence data. In this frame, the information generated by HRMS identification of novel compounds can be added to parent toxins libraries, and further used for retrospective analysis of full-scan data.

This can usefully concur to acquire qualitative occurrence data for better food quality controls, and more importantly, to ensure food safety.

Besides the advantages described above, HRMS still presents some critical points to be considered for its application in contaminants analysis. Among the possible disadvantages, the cost of instrumentation and the difficulties in big data management are often reported, but the improvement of software and storage systems as well as the introduction on the market of bench-top instruments favor broader diffusion LC-HRMS based methodologies.

However, from a chemical point of view, issues such as isobars co-elution, and unknown molecule identification have to be solved to allow an effective use of HRMS for food safety purposes. The innovative technique of ion mobility spectrometry (IMS) can be used to ascertain complementary information about analytes, adding a third dimension of separation based on size, shape, and charge of ions. The coupling of the two strategies, IMS and LC-HRMS platform, may act as a powerful tool to (i) improve the quality of mass spectral information obtained thanks to a filtered background; (ii) to increase the peak capacity to separate isomeric/isobaric compounds which can neither be resolved by MS and sometimes nor by UHPLC; (iii) to enhance analyte identification thanks to structural information (size and shape) based on collision cross-section measurement.

## 7. The Potential Benefits of Ion Mobility Mass Spectrometry in Mycotoxin Analysis

In the first section, the many possibilities of HRMS as a tool for modified mycotoxin analysis were reviewed. However, it should be stated that confirmation and structure elucidation of new unknown molecules and the unambiguous identification of isomers still remains challenging when using conventional mass spectrometry methods.

In comparison, NMR spectroscopy has been successfully employed with the goal of recognizing modified mycotoxins structure [22,60]. The NMR-based approach is efficient for evaluating isomeric heterogeneity, and for structural elucidation, but has the limitation of needing a considerable amount of analytes and obtaining a single molecular species following the purification steps.

Ion mobility spectrometry (IMS) is a promising approach that can overcome the above mentioned HRMS and NMR limitations, making it an ideal candidate for improving confidence in the identification and separation of structurally closely related isomers. IMS is a gas-phase electrophoretic technique that provides a new dimension (3D) of separation based on size, shape, and charge of ions. The ion mobility spectrometer consists of four main components that can be identified as: the sample introduction system (SIS), the ionization source, the drift tube region for separation and selection of ions, and the detection area [61,62]. In this review we mainly focus on the separation region, detailed information on the other components can be found elsewhere [61]. Once ionized, ions are directed to the drift tube region that contains an electric field, drift gas separates them according to their mobility. Ions moving in a gas phase medium and in the presence of an electric field are accelerated due to coulomb forces and slowed down due to collisions with molecules of the gas medium [61]. Thus small and compact ions travel faster and will reach the detector before large and heavy ions. In this way isobars are separated in the mobility spectrum, where the ion current is plotted as a function of the drift time or the compensation voltage [50].

Eight different types of IMS have been recently reviewed [61]. However, it should be noted that not all IMS devices are stand-alone instruments and for the purpose of the present review, only the IMS hyphenated with a mass spectrometer are discussed. A common hyphenated technique includes coupling IMS with MS (IMS-MS) in which IMS works as a pre-filter by confirming ion identities for the MS system. In addition, since IM separation typically occurs in a millisecond timeframe and MS detection, in a typical TOF instrument, takes only microseconds, additional separation techniques such as liquid chromatography (LC) can be hyphenated without compromising the speed of MS detection. So far, four major IMS-MS separation approaches are currently commercially available coupled with MS: drift-time IMS (DT-IMS) [63], traveling-wave IMS (TW-IMS) [64,65], high field asymmetric waveform IMS (FAIMS) [66], also known as differential-mobility spectrometry (DMS), and trapped IMS (TIMS) [67,68,69].

In DTIMS and TWIMS all the ions pass through the mobility cell ions and are separated based on the time it takes to traverse the cell. Such devices are generally used for untargeted screening experiments. FAIMS/DMS devices separate ions by varying voltages, filtering ions in a space-dispersive fashion. TIMS-MS separates ions based upon differences in mobility, after trapping and selectively ejecting them. Readers interested in the principles behind these IMS technologies can refer to previous reviews [70].

Applications in the food analysis field, especially when analyzing contaminants, have been mainly addressed applying FAIMS technologies [71,72,73] probably because of the advantages offered by the filtering effect as well as for the possibility of the device being moved and placed at the front-end of the mass spectrometer. On the other hand, TWIMS applications are rapidly growing [74,75,76] because, enabling CCS values measurement, it has found application as both a separation device and a structural elucidation tool.

Considering the complexity of food and feed samples, the use of LC-IMS-MS hyphenated methods is starting to be considered vital for versatile applications. Indeed, LC-IMS-MS potentially provides three major benefits to modified mycotoxins detection compared to traditional approaches. First, LC-IMS-MS improves the peak capacity and signal-to-noise ratio of traditional analytical approaches providing cleaner mass spectra obtained from the filtered background [71,77,78]. Second, it allows the separation of co-eluting isobaric metabolites according to their mobility [79], simplifying the interpretation of mass spectra. Third, IMS enhances confidence in analyte identification thanks to the measurements of the collision cross section (CCS) [80], a physicochemical measure related to the conformational structure of ions (size, shape, and charge).

### 7.1. Peak Capacity and Signal-to-Noise Ratio Improvement

The coupling of IM with liquid chromatography (LC) and HRMS gives a degree of orthogonality to both techniques by separating co-eluting LC compounds in mobility space before mass analysis. Hence, the overall peak capacity of the method is increased [81,82] making IM-MS highly suitable for food safety control analysis. In this frame, a broad range of food contaminants such as herbicides [83], pesticides [75,84], mycotoxins [71,77,78], and veterinary drug residues [85] have been successfully detected by IMS.

Although mycotoxins have been scarcely analyzed by IMS-MS, three applications using low resolution mass spectrometry have been reported so far [71,77,78]. An unusual type of IMS, corona discharge ion mobility spectrometry (CD-IMS) was applied to determine aflatoxin B1 (AFB1) and B2 (AFB2) in pistachio samples aiming to monitor spoilage status [77]. Sample extracts were directly introduced into the corona discharged ionization region via a headspace (HS) device without any chromatographic separation. As expected by the authors, IMS was not able to distinguish between AFB1 and AFB2 due to their similar chemical structure and their very close molecular weight. Thus they measured the total AFBs since it was demonstrated that their IMS response factors were identical [77]. The resulting LOQ and LOD (0.5 and 0.1 ng·mL^−1^, respectively) were in line with those reported in literature obtained using different chromatographic and spectrometric techniques. In addition, pistachio samples were analyzed to demonstrate the capability of the method in detecting aflatoxins in real samples. The same authors applied the proposed approach some years later [78] for the analysis of ochratoxin in licorice roots. Following extraction and purification by passage through an immuno-affinity column, the achieved LOD in real samples was compliant with established concentration limits for licorice roots (20 µg/Kg).

A significant improvement of the detection limits was also measured for ZEN and its metabolites α-ZEL, β-ZEL, and β-zearalanol (ZAL) in maize using FAIMS technology and direct infusion or flow injection [71]. In fact, compared to ESI-MS or ESI-MSMS, a five-fold improvement in the signal to noise ratio was reported. This result was attributed to the ability of FAIMS equipment to operate as an ion filter, focusing ions and reducing the chemical background attributable to the matrix. The achieved LODs for ZEN, α-ZEL, β-ZEL, and β-ZAL were 0.4 ng·mL^−1^, 3.2 ng·mL^−1^ and 3.1 ng·mL^−1^, respectively. Thus, FAIMS filter step prior to ESI-MS analysis was able to selectively resolve and quantify species that otherwise cannot be selectively analyzed by ESI-MS alone. Additionally, since analytes are separated on account of their compensation voltages, reducing the time required for each sample run to about 1 min, the authors suggested that FAIMS might allow overstepping of the chromatographic separation [71].

LC-ESI-FAIMS was also used to develop a quantitative method for the determination of marine toxins in mussel tissue [73]. They investigated in depth how to improve the method sensitivity in relation to the number of CV values monitored at a given time. In fact, one of the limiting factors for analytical sensitivity is the duty cycle of the FAIMS device, which has a switching time between different CV values of about 100 ms, the time required to empty the device of ions that experience a particular CV. This means that limiting the number of CV values simultaneously monitored is an effective way of limiting sensitivity losses observed when using FAIMS in combination with LC. Two approaches were investigated for limiting the number of monitored CVs. The first one was developed using time periods with a limited number of optimized CV values at a given retention time, the second one reducing the number of monitored CVs to three values, which provided coverage of all analytes close to, but not at their optimal CV. The latter method proved to be more robust but less sensitive due to the fact that toxins were not detected at their optimum CV values, but was more suitable for analysis of large sample sets where RT could be expected to drift slightly.

The above mentioned IMS-MS approaches are pioneering, since they represent the first application of ion mobility spectrometry in the mycotoxin field. On the whole, the achieved limits of detection for these applications were in agreement with those required from the regulatory authorities, and confirming the method applicability to real samples and then the fitness for purpose, led to the expectation that IMS may help trace analysis control compliance. Thus, the development of further IMS-MS methods to extend the number of monitored parent and modified mycotoxins are encouraged.

**Figure 4:**
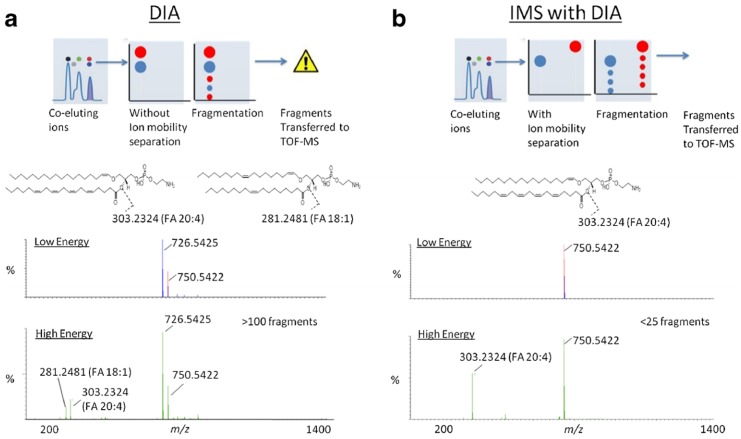
MS and MS/MS cleaner mass spectra obtained by using LC-IM-QTOF (**b**) compared to those obtained by LC-QTOF (**a**) (from Paglia et al. [86]).

In this regard, more recently, a novel approach to screening multi-class pesticides by TW ion mobility time-of-flight mass spectrometry detection was successfully developed [75]. The authors demonstrated that combining full scan and mobility XIC (extracted ion chromatogram) it was possible to detect the mass spectrum of indoxacarb, that was masked by other co-eluting compounds in the scan spectra. This example demonstrated how drift times give a higher level of selectivity to the overall method as no interfering compound resulted at the same retention time, drift time, and measured exact mass. In addition, once the pesticide has been identified using its retention time, exact mass, and drift time, the resulting cleaned mass spectrum facilitates the identification process. In line with these findings, the cleaning effect due to ion mobility separation on MS/MS spectra was demonstrated [86]. Applying the DIA mode for the MS analysis of complex extracts could result in MS/MS spectra containing a mixture of product ions derived from co-eluting precursors, complicating interpretation of the spectra and the overall identification process (Figure 4). Combining IMS-MS with DIA might allow the separation of co-eluted precursor ions before fragmentation, resulting in a drift-time correlation of product ions with their respective precursor ions and thus cleaner MS/MS product-ion spectra.

This phenomenon might offer a straight benefit when analyzing mycotoxins in complex food and feed matrices and in particular modified mycotoxins, considering that they are usually present at a low concentration level. Despite the HRMS improvement, quite significant discrepancies when comparing relative intensities of fragment ions measured in pure solvent with those measured in matrix were reported [31]. Thus, these clean MS and MS/MS spectra might, in turn, facilitate compound identification and reduce false-positive assignments in complex food matrices.

### 7.2. LC-IMS-MS Enables the Separation of Isobar Molecules

Filtering out interferences, LC-IMS-MS may also allow the separation of co-eluting isobars and isomers that are difficult to separate by traditional LC-MS. Isobar co-elution may occur when expected unknowns are tentatively identified by LC-HRMS, as described in some examples reported in the previous sections. This issue is of a great relevance for mycotoxin analysis, above all when co-eluting modified forms differ in their toxicological profile, as for the acetylated derivatives of DON. With regard to intestinal toxicity, 3Ac-DON was found to be less toxic than DON, which was less toxic than 15Ac-DON [87]. As a result, a precise quantification of the different isoforms has to be performed. However, considering that they differ only in the position of the acetyl group and thus similar polarities of these two mycotoxins, it has not been possible to achieve chromatographic separation so far [88]. Hence, different strategies have been developed to reach a correct quantification. By MS-single quadrupole detection, Biancardi et al. [89] calibrated the response for 15Ac-DON and 3Ac-DON separately and reported the results as the sum of 3- and 15Ac-DON (Ac-DONs). Afterwards, thanks to the selective detection power of MS/MS, separate identification was performed due to the difference in characteristic daughter ions in MRM mode, identified as *m*/*z* 339.5 >137.2 and 339.5 >321.1, and *m*/*z* 339.5 >213.0 and 339.5 >230.9 for 15Ac-DON and 3Ac-DON, respectively [88]. In a recently published multi-mycotoxin method [15], the two acetylated derivatives were detected by taking advantage of the detection polarity. In fact, 15Ac-DON was detected in positive mode (*m*/*z* 356.1, [M+NH_4_]^+^) and 3Ac-DON in negative mode as acetate adduct (*m*/*z* 397.1, [M+CH_3_COO]^−^).

Hence, it is evident that the potential of LC-IMS-MS to enhance isomer separation, would overcome challenges associated with modified mycotoxin isomers analysis that will not otherwise be achieved. In addition, the proper separation and then quantification of 3-Ac-DON and 15-Ac-DON is essential in order to collected further data and better characterize their potential contribution to the total exposure to DON.

Even more challenging is the case of modified mycotoxins, whose analytical standards are not available, since the traditional quantification methods, represented by tandem MS, are not applicable. The detection and identification of these unknown modified forms is permitted by taking advantage of the accurate mass and the isotopic pattern distribution provided by HRMS. However, no information about putative co-eluting isomers having the same *m*/*z* value can be obtained with only LC-HRMS. When analyzing oligo-glycosides mycotoxins, indeed, information about binding position and configuration (α or β) between the sugar moiety and the mycotoxin, and the linkages, α/β1-4, α/β1-6, cannot be achieved. Regarding binding configuration, information available in the literature is quite contradictory. McCormick et al. [60] reported the occurrence of both T2-α-Glc and T2-β-Glc in naturally contaminated wheat and oat samples. By contrast, Meng-Reiter et al. [48], stated that since the UDP-glucosyltransferase is an inverting enzyme, the detection of the α-glucosyl isomer should be unexpected. In agreement, Zachariasova et al. [38] reported an increase of free DON after DON oligoglucosides incubation with fungal β-glycosidase, that was obviously caused by its release from the β-bound forms. However, also stating the glucosylic bound for DON-oligo-Glc, the number of possible theoretical isomeric structures was higher than the number of chromatographic peaks detected (see Figure 2 and Figure 3) [38]. Thanks to the enhancement in isomer separation offered by applying DTIMS coupled with quadrupole time-of-flight spectrometer (Q-TOF), one more DON-di-Glc and two more DON-tri-Glc peaks separated by their drift time were detected [79]. These additional peaks could be due to the linkages, 1–4 or 1–6, between the sugar moieties and the mycotoxin, since the bounding position was confirmed by HRMS-MS/MS. Therefore, IMS-MS allowed the detection and subsequently the characterization of new isomeric DON-oligo-Glc, also increasing confidence in results.

The same approach could be applied to resolve other modified mycotoxins such as olygo-glycosides forms of ZEN or α/β ZEL, that have already been detected [38] but whose structures have not yet been elucidated, giving new insight into the mycotoxin biotransformation that may occur in plants and/or animals.

It is evident from the examples given that a strong synergy arises between IMS and MS. IMS-MS can act as a tool to separate complex mixtures, to resolve ions that may be indistinguishable by mass spectrometry alone. This is vital in the modified mycotoxins field, since many different types of isomers (diastereoisomers, epimers, anomers, protomers) may occur and need to be separated to achieve a correct quantification and subsequently to perform a reliable risk assessment. As for ADON isomers, also α/β-ZEL diastereoisomers present different toxicity. α/β-ZEL may undergo phase I and phase II metabolism [47], both in plants and in humans, leading to a wide range of metabolites having different configurations and potentially different toxicities. Thus, the separation and then elucidation of the binding configuration of the conjugated metabolites is highly advisable also for the toxicological outlook.

### 7.3. CCS Value: A New Unambiguous Molecular Descriptor

In addition to signal-to-noise improvement and enhancement in separation of co-eluting isobar molecules, IMS-MS has also been applied to more high-throughput analytical approaches for confirming compound identity, providing molecular structural and conformational information.

Once the drift time is recorded, this can be converted into a CCS value, which represents the effective area for the interaction between an individual ion and the neutral gas through which it is travelling. Thus CCS is an important distinguishing characteristic of an ion in the gas phase and, being related to its chemical structure and three-dimensional conformation, can provide specific information on ionic configuration and potential structural confirmation.

Nowadays, CCS values can be routinely measured as an integrated part of the LC-HRMS experiment. In DTIMS instruments, CCS can be directly derived from the drift time. In TWIMS [90,91,92] instruments, CCS can be experimentally derived by using IMS calibration performed using compounds of known CCS under defined conditions (i.e., gas type and pressure, travelling wave speed or height). This allows CCS to be used alongside the traditional molecular identifiers of precursor ion accurate mass, fragment ions, isotopic pattern, and retention time as a confirmation of compound identity [93]. Indeed, Goscinny and colleagues [75] developed a TWIMS approach to screening multi-class pesticides and suggested the inclusion of the pesticide CCS values as a new identification point (IP) (Commission Decision 2002/657/EC [94]), to increase confidence in the results. Overall, they measured CCS values for 150 pesticides, using standard solutions, building an in-house CCS library with associated retention times, accurate masses, and diagnostic fragments. Thus, the CCS value may be included in the contaminants screening workflow, as reported in Figure 1, and it can be used as an additional means of filtering the screening data to significantly reduce the proportion of false positive and false negative detections. Therefore, the CCS tolerance of ±2% in combination with the traditional confirmation threshold filters of *m*/*z* (±5/10 ppm) and retention time (±2.5%) will lead to a more definitive identification of the species of interest. A further dataset providing CCS values for 200 pesticides has been recently reported by Regueiro et al. [84].

Population of databases with CCS values for pesticides and mycotoxins is pivotal in order to support the inclusion of CCS values as a new identification point (IP).

In addition, since CCS measurements are undertaken in the gas phase, remotely from the ion source, their values are not affected by sample matrix and are consistent between instruments and across a range of experimental conditions [86,93]. Taking into account the analytical effort made in recent years for validating extraction and detection procedure depending on the sample matrix, the great advantage offered by the CCS measurement is evident. Moreover, it has been demonstrated that the concentration of the compound had no significant effect on the drift time values and thus on the CCSs [75]. This will help in avoiding false negative assignments in the screening confirmation procedure, mainly when analyzing contaminants close to method LOQs, since matching with HRMS/MS in libraries could be hard due to the low intensity of the fragmentation pattern [31].

In agreement, Paglia and co-workers [95] also confirmed the high reproducibility of CCS measurements of lipids classes in varying matrices. They created a CCS database for lipids that includes 244 CCS values, aiming to implement the ion-mobility derived CCS in routine lipidomics workflow.

These findings raised the possibility that CCS can be used to help the identification process of targeted compounds, and, as for pesticides and lipids, CCS can be inserted in a routine workflow for parent and modified mycotoxin screening and used as an identification parameter. Data bases of mycotoxins can be created using CCS obtained by running standard compounds, providing an additional coordinate to support mycotoxin identification, and reducing the number of false positive and false negatives of the targeted analysis. In addition, at the end of the untargeted screening process (see Figure 1), CCS can be used to confirm the structure of expected unknowns by matching the theoretical and the experimental CCS values.

In TWIMS devices, poly-DL-alanine is often used as IMS calibrant for deriving CCS. Since peptides have unique physical properties and gas-phase conformations, it might not be ideal to calculate the accurate CCS values for all metabolites and lipids classes. For instance, alternative calibrants have been proposed for specific lipids, which better reflect their chemical structure [96].

CCS values may also be estimated computationally if the 3D structure is known. A comparison of the theoretical and experimentally derived collision cross-sections can be utilized for the accurate assignment of isomeric metabolites. Recently, the CCS areas were used to elucidate the α and β epimeric forms of glycosylated T-2 and HT-2 toxins [80]. The two isomeric forms had already been separated by UHPLC-MS/MS [48] however, thanks to additional information provided by the CCS value, it was possible to confirm the bounding configuration between the toxin and the sugar moiety [80].

The aforementioned application is the only one developed so far in the field of mycotoxins exploiting CCS potential; however IMS-MS, as a tool to gain insight into structural information, would expect to rise rapidly, offering a unique means of characterization. New modified forms, i.e., expected unknown mycotoxins, may be discovered and unequivocally characterized by matching theoretical and experimental rotationally averaged cross-sectional areas, despite the lack of analytical standards. Regarding unexpected unknown mycotoxins, even though HR-IMS reduces the number of possible candidates due to accurate mass, isotopic pattern, MS/MS fragment ions, and CCS values, the identification might still be challenging. In these particular cases, the use of NMR still represents the only approach for identification*.*

## 8. Conclusions and Future Trends

In the last few years, a significant increase in the number of studies reporting HRMS-based approaches for food contaminant analysis has been reported. The analytical potential of high resolving power, accurate mass, and acquisition in full scan permits a retrospective analysis using extensive databases of hundreds of analytes and enabling the investigation of ‘newly discovered’ mycotoxins in the data of prior-analyzed samples. Therefore, HRMS is undoubtedly going to redefine LC-MS workflow since targeted and routine quantification as well as qualitative research analysis can be performed with the same instrument. This situation is also facilitated by the launch from many MS companies of the latest generation of HRMS instrumentation designed for routine analysis and equipped with user-friendly dedicated data processing software.

On the other hand, ion mobility spectrometry is starting to be successfully employed in mycotoxin trace analysis with the aim of increasing signal-to-noise ratio, gaining higher sensitivity, and with longer dynamic range [71,77,78]. However, applications of IMS in separation and structure confirmation of mycotoxins has not been explored adequately so far, even though it offers great potential for gaining insight into the formation and characterization of new modified forms. In particular, the CCS values may be added in targeted and untargeted screening workflow, providing an additional coordinate to support mycotoxin identification, reducing the number of false positive and false negatives and confirming the structure of expected unknowns. In conclusion, all evidence points towards future growth in the number of applications of HRMS and HR-IMS in food safety, as the power of this technique gains wider recognition.

## Figures and Tables

**Table 1:** Examples of parent and modified mycotoxins classified in known/unknown categories, according to the above mentioned definition.

Known/Unknown Categories	Modified Mycotoxin	Matrix	MS Equipment	Identification Based on	Analytical Standard
Expected knowns	Aflatoxin M1	Cheese [39]	Q-Trap	authentic standards	commercially available
	DON3Glc	Wheat and maize [22]	Q-Trap	authentic standards	commercially available
	3/15Ac-DON	Wheat [23]	Q-Trap	authentic standards	commercially available
Unexpected knowns	Aflatoxin M1	Feed [40]	QqQ	authentic standards	commercially available
	enniatins, alternaria toxins, T-2/HT-2 toxins	Dietary supplements [41]	Q-Trap	authentic standards	commercially available
	FB2	Culture media [42]	QqQ	authentic standards	commercially available
Expected unknowns	T2-Glc	Wheat and oats [43]	LTQ Orbitrap	HRMS	in-house synthesized
	15Ac-DON-Glc	Wheat [44]	LTQ Orbitrap XL	authentic standards	in-house synthesized
	DON-oligoglycoside	Malt and Beer [38]	Exactive Orbitrap	HRMS	n.a.
	NIV-Glc	Wheat [45]	LTQ Orbitrap	HRMS/MS	n.a.
	Desmethyl Enn B1	Human liver [46]	Q-Tof	HRMS/MS	n.a.
	ZEN-MalGlc	Wheat [47]	Q-trap	MS/MS	n.a.
Unexpected unknowns	Feruloyl-T2	Barley [48]	Exactive Plus Orbitrap	HRMS/MS	n.a.
	DON-2H-glutathione	Wheat [44]	LTQ Orbitrap XL	HRMS/MS	n.a.
	Pentahydroxyscirpene (PHS)	Barley [49]	Q-Tof	MS/MS	in-house synthesized
	DON-3-Glc lactone	Wheat [50]	Exactive Orbitrap	HRMS	n.a.

Table columns: MS equipment = mass spectrometry instrument; identification based on = mycotoxin identification based on matching retention time, *m*/*z* and MS/MS fragment with that of authentic standards, or based on accurate mass (HRMS) and accurate fragments (HRMS/MS); analytical standard = mycotoxin standard commercially available, not available (n.a.) or in-house synthesized by research group.

**Table 2:** Summary of calculated exact masses for putative phase I and phase II mycotoxin modifications.

Modification	Mass Change (Da)	Molecular Formula Change
Hydrogenation	2.0151	H_2_
Hydroxylation	15.9944	O
Methylation	14.0151	CH_2_
Acetylation	42.0100	C_2_H_2_O
Glycine	57.0209	C_2_H_3_NO
Sulfate	79.9563	SO_3_
Sulfonation	102.9460	SO_3_Na
Ferulic acid	176.0468	C10H_8_O_3_
Cysteine	119.0036	C_3_H_5_NO_2_S
Acetyl-cysteine	161.0141	C_5_H_7_NO_3_S
Glucose	162.0523	C_6_H_10_O_5_
Cysteine-glycine	176.0250	C_5_H_8_N_2_O_3_S
Glucuronic acid	176.0315	C_6_H_8_O_6_
Acetyl-glucoside	203.0550	C_8_H_11_O_6_
Malonyl glucoside	248.0527	C_9_H_12_O_8_
Glutathione	305.0682	C_10_H_15_N_5_O_6_S
Di-glucoside	324.1051	C_12_H_20_O_10_
Malonyl di-glucoside	410.1055	C_15_H_22_O_13_
Tri-glucoside	486.1579	C_18_H_30_O_15_
Di malonyl-di glucoside	497.1137	C_18_H_25_O_16_
Tetra-glucoside	648.2107	C_24_H_40_O_20_
